# Amelioration of age‐related brain function decline by Bruton's tyrosine kinase inhibition

**DOI:** 10.1111/acel.13079

**Published:** 2019-11-17

**Authors:** Akang E. Ekpenyong‐Akiba, Marta Poblocka, Mohammad Althubiti, Miran Rada, Diana Jurk, Sandra Germano, Gabriella Kocsis‐Fodor, Yu Shi, Juan J. Canales, Salvador Macip

**Affiliations:** ^1^ Mechanisms of Cancer and Aging Laboratory Department of Molecular and Cell Biology University of Leicester Leicester UK; ^2^ Department of Biochemistry Faculty of Medicine Umm Al‐Qura University Mecca Saudi Arabia; ^3^ Ageing Research Laboratories Institute for Ageing and Health Newcastle University Newcastle upon Tyne UK; ^4^ Centre for Integrated Systems Biology of Ageing and Nutrition Institute for Ageing and Health Newcastle University Newcastle upon Tyne UK; ^5^ Department of Physiology and Biomedical Engineering, Robert and Arlene Kogod Center on Aging Mayo Clinic Rochester MN USA; ^6^ Division of Psychology School of Medicine University of Tasmania Hobart TAS Australia

**Keywords:** BTK, cellular senescence, healthspan, p53, progeria

## Abstract

One of the hallmarks of aging is the progressive accumulation of senescent cells in organisms, which has been proposed to be a contributing factor to age‐dependent organ dysfunction. We recently reported that Bruton's tyrosine kinase (BTK) is an upstream component of the p53 responses to DNA damage. BTK binds to and phosphorylates p53 and MDM2, which results in increased p53 activity. Consistent with this, blocking BTK impairs p53‐induced senescence. This suggests that sustained BTK inhibition could have an effect on organismal aging by reducing the presence of senescent cells in tissues. Here, we show that ibrutinib, a clinically approved covalent inhibitor of BTK, prolonged the maximum lifespan of a *Zmpste24^−/−^* progeroid mice, which also showed a reduction in general age‐related fitness loss. Importantly, we found that certain brain functions were preserved, as seen by reduced anxiety‐like behaviour and better long‐term spatial memory. This was concomitant to a decrease in the expression of specific markers of senescence in the brain, which confirms a lower accumulation of senescent cells after BTK inhibition. Our data show that blocking BTK has a modest increase in lifespan in *Zmpste24^−/−^* mice and protects them from a decline in brain performance. This suggests that specific inhibitors could be used in humans to treat progeroid syndromes and prevent the age‐related degeneration of organs such as the brain.

## Graphical abstract

We showed that blocking Bruton's tyrosine kinase (BTK) dampens p53 activity and the induction of senescence in vitro. Here, we show that BTK inhibitors increase maximum lifespan and healthspan of progeroid mice. Moreover, they prevent the deterioration of certain cognitive functions and reduce the accumulation of senescent cells in the brain. This suggests that clinically available BTK inhibitors could ameliorate some features of ageing in susceptible patients.

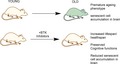

## 1 INTRODUCTION

Aging is associated with a time‐dependent functional decline induced by the interaction of several mechanisms (Lopez‐Otin, Blasco, Partridge, Serrano, & Kroemer, 2013). This process increases the susceptibility to various degenerative pathological conditions, leading to high morbidity rates and, eventually, death (Campisi, Andersen, Kapahi, & Melov, 2011). Indeed, the incidence of many diseases, such as atherosclerosis, osteoporosis, arthritis, hypertension, Alzheimer's and cancer, increases with age (Munoz‐Espin & Serrano, 2014). This link between aging and debilitating ailments has fuelled the search for anti‐aging interventions.

Although not all the causal events have been fully characterized, it has been proposed that the main determining factors of aging can be summarized into nine groups: genomic instability, telomere attrition and telomere damage, epigenetic alterations, loss of proteostasis, deregulated nutrient sensing, mitochondrial dysfunction, accumulation of senescent cells, stem cell exhaustion and altered intercellular communication, collectively known as the hallmarks of aging (Lopez‐Otin et al., 2013). Efforts to slow down age‐related dysfunctions are currently being aimed at preventing one or more of these events.

Of all these hallmarks, senescent cells have been recently gaining prominence as a promising anti‐aging target. Senescent cells disrupt tissue homeostasis by secreting factors that promote inflammation, tumour growth and tissue damage, and this leads to a functional decline and age‐related symptoms (Tchkonia, Zhu, Deursen, Campisi, & Kirkland, 2013). Moreover, senescent cells are a contributing factor in the development of pulmonary fibrosis, bronchiectasis, osteoarthritis, anxiety, liver steatosis and atherosclerosis, among other chronic diseases (Munoz‐Espin & Serrano, 2014). Recent evidence has shown that eliminating senescent cells leads to prolonged lifespan and time lived in full health (healthspan) in mammalian models (Baker et al., 2016, 2011; Xu et al., 2018), thus confirming that the increased presence of senescent cells in tissues has an important role in aging phenotypes. This provides a rationale for devising therapeutic strategies to prevent a time‐dependent buildup of senescent cells in human tissues, which could potentially ameliorate the symptoms of aging as well as prevent age‐related pathologies.

In order to devise novel strategies to detect senescent cells, we performed a screening of the senescent surfaceome, those proteins present in or associated with the plasma membrane of senescent cells (Althubiti et al., 2014). We identified several new specific markers, including the Bruton's tyrosine kinase (BTK), a nonreceptor tyrosine kinase that belongs to the Tec family and is essential for B‐cell maturation (Mohamed et al., 2009). BTK is located at the cell membrane but it can also be found in the nucleus, and it has been shown to have pro‐apoptotic and tumour suppressor functions (Ta et al., 2010). In B cells, BTK is activated after an antigen binds to the B‐cell receptor, which leads to its phosphorylation at tyrosine 551 by SRC family kinases and its autophosphorylation at tyrosine 223 (Rawlings et al., 1996). A pathological BTK upregulation has been shown in B‐cell malignancies such as chronic lymphocytic leukaemia (Herman et al., 2011).

We have reported that BTK is a crucial part of the p53 pathway, where it acts as a modulator of p53 activity (Althubiti et al., 2016). We found that BTK is expressed in response to damage and induces phosphorylation of the N‐terminus of p53, which leads to increases in protein levels and activity. Also, BTK binds to and phosphorylates MDM2, mediating a loss of ubiquitination activity and further stabilizing p53 (Rada et al., 2017). BTK has a similar effect on p73, a p53 family member that also acts as a tumour suppressor (Rada, Barlev, & Macip, 2018a). Inhibition of BTK interferes with the upregulation of p53 target genes, which lead to a severe impairment in the induction of p53‐mediated cell fates, especially senescence (Althubiti et al., 2016). This defines a novel role of BTK in tumour suppression (Rada, Barlev, & Macip, 2018b).

Given this important regulatory role of BTK in p53‐induced senescence, we reasoned that blocking BTK activity for prolonged periods of time could prevent the accumulation of senescent cells in vivo and thus diminish some of the deleterious effects of aging. Here, we show that chemical inhibitors of BTK can indeed increase lifespan and healthspan in animal models of premature aging. Of particular interest is the preservation of certain cognitive functions in mice when BTK is chronically inhibited. We propose that BTK inhibitors could be a clinically relevant strategy to protect humans from age‐related loss of fitness, especially when a cognitive decline is anticipated.

## 2 RESULTS

### 2.1 BTK inhibition reduces the impact of aging in progeroid mice

**Figure 1:**
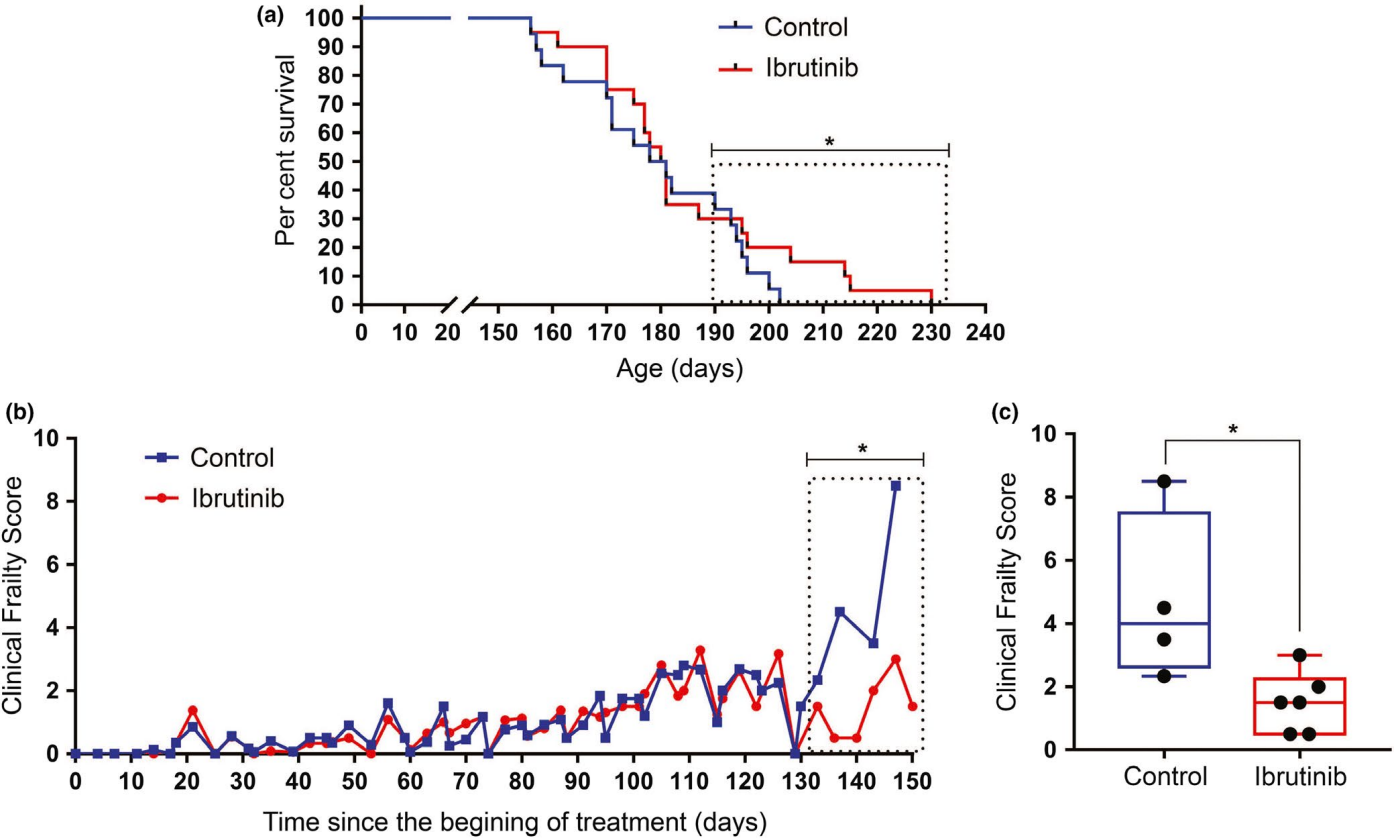
Ibrutinib effects on lifespan and healthspan of progeroid mice. (a) Kaplan–Meier survival curves for control and ibrutinib‐treated *Zmpste24^−/−^* mice. All treated mice in this figure were given 10 mg/kg ibrutinib. The median lifespan of control and treated mice did not differ significantly (*p* = .2813) by the log‐rank (Mantel–Cox) test. However, survival in the boxed area, corresponding to the longest lived mice (top third of each cohort), showed a statistically significant difference (*p* = .0144). *n* = 18 (control) and 20 (ibrutinib). (b) Clinical Frailty Score in the same experiment. See Table S2 for parameters used. Each parameter is scored as absent (0), mild (0.5) or severe (1), and the total sum of values for each mouse is recorded. *n* = 18 (vehicle) and 20 (ibrutinib). Data are presented as the mean of the values, and statistical analysis of the boxed area is done using an unpaired *t* test. *p = .0248. (c) Late life clinical frailty scores of the boxed area in (b), presented in a box and whiskers plot showing median (bar), upper and lower quartiles (box) and lowest and highest values (error bars). Each dot represents one mouse. *p = .0248

We have previously shown that blocking BTK hampers p53‐induced senescence in vitro (Althubiti et al., 2016). We reasoned that BTK inhibition could thus prevent accumulation of senescent cells in vivo. According to recent reports (Baker et al., 2016, 2011), this could lead to an amelioration of age‐related conditions and an increased lifespan. To test this possibility, we used ibrutinib (PCI‐32765), an inhibitor that irreversibly binds to the C481 residue of BTK and inhibits Y223 stimulation and has been approved to treat mantle cell lymphoma and CLL (Aalipour & Advani, 2014). We studied its effects on *Zmpste24^−/−^* mice, which display a premature aging phenotype with an average maximum lifespan of 8 months (Pendas et al., 2002), mediated at least in part by a pathological increase in p53 signalling (Varela et al., 2005). Mice were dosed with 10 mg/kg ibrutinib continuously (twice a week by oral gavage) from 2 months of age up to 8 months (or when the humane end points were reached, as described in Table S1). As hypothesized, this leads to a reduction in the accumulation of senescent cells in different tissues, as measured by the expression of several senescent markers by Western blot and qPCR (Figure S1a,b). Although we did not observe any changes in the average lifespan of treated mice when compared to controls (Figure 1a), the maximum survival was increased (from 202 to 230 days). This was accompanied by a statistically significant difference in survival in the longest lived mice (boxed area). When these mice were analysed for signs associated with frailty (Whitehead et al., 2014; Table S2), we observed that there were no differences in the first 4 months after initiating treatment, during which the scores were low for both control and treatment groups (Figure 1b). However, health started to deteriorate in the control mice after that point, whereas it was preserved in the treated animals (Figure 1b,c). Of note, none of the treated animals had important side effects (see Table S2). There were no visible tumours in these mice, which could have resulted from the inhibition of tumour suppressors such as p53, and no cancers were observed in necropsies (data not shown). These results show that a prolonged ibrutinib treatment can ameliorate aging in progeroid mice by modestly increasing lifespan, generally reducing frailty and significantly extending their maximum lifespan.

### 2.2 Expression of BTK in brains of aged mice

**Figure 2:**
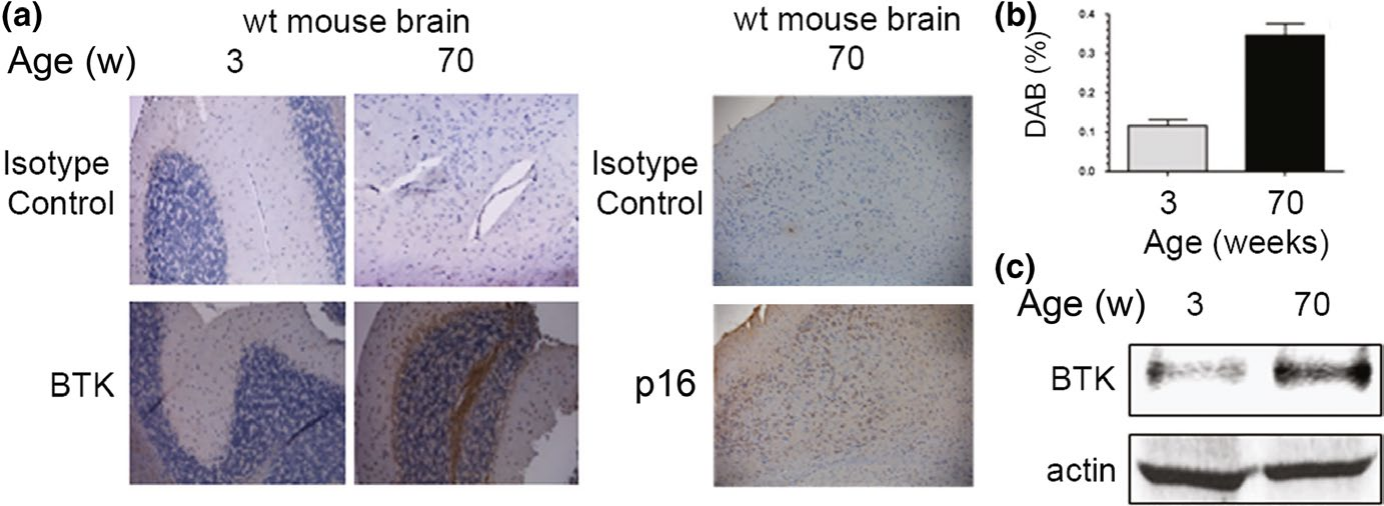
BTK expression is increased in aged mouse brains. (a) Representative images of immunohistochemical staining of wild‐type young (3 weeks) and old (70 weeks) mice brains using a BTK antibody and an isotype control. (b) Quantitation (percentage of DAB) of samples in (a), taken from three mice per group (six pictures taken from each animal). Bars show average values, and error bars represent standard deviation. (c) Representative Western blot of whole brain lysates from the same mice, showing the levels of expression of BTK and β‐actin (loading control)

Given the fact that BTK expression is unlikely to be homogenous in all aged tissues, we hypothesized that the effects of BTK inhibition in ameliorating age‐related symptoms would likely vary between different organs. In an initial screen of different normal mouse tissues (Figure S1c), we observed that BTK expression increased substantially in different organs of old wild‐type mice, including brain (Figure 2a,b). This was concomitant with the expression of p16, a known marker of senescent cells (Baker et al., 2011). Also, the average BTK protein expression in the aged brain was also increased when compared to young counterparts, as measured by Western blot (Figure 2c). This indicates that mouse brains significantly upregulate BTK expression over time and suggests that this tissue could be particularly sensitive to the BTK inhibitors in the context of aging.

### 2.3 Reduction in senescent cells in the brain of progeroid mice after BTK inhibition

**Figure 3:**
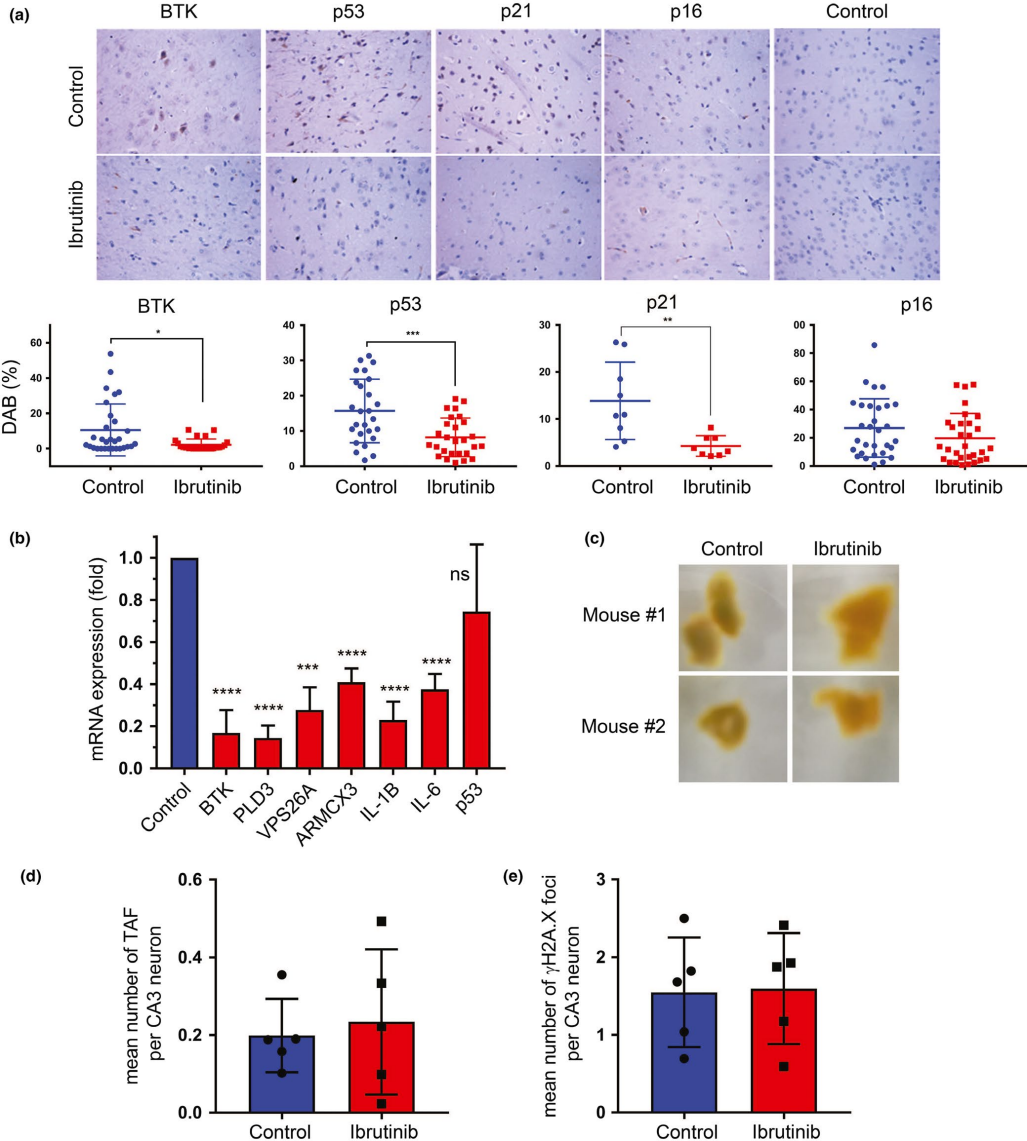
Ibrutinib reduces the accumulation of senescent cells in the aging brain. (a) Representative images of immunostaining of brain of control and 10 mg/kg ibrutinib‐treated *Zmpste24^−/−^* mice, 121 days after the beginning of treatment (196 days old), using antibodies against BTK and other known markers of senescence (p53, p21, p16), compared with an isotype control. Magnification: 40×. Graph shows quantitation of 8–15 animals per group (and 1–6 pictures taken from each animal). Lines show mean values of the percentage of DAB, and error bars show standard deviation. Each dot represents one sample. *p < .03; **p < .003; ***p < .0003 (unpaired *t* tests). (b) mRNA expression levels of different senescent markers as measured by quantitative real‐time PCR of samples from the ibrutinib‐treated *Zmpste24^−/−^* mice, normalized to the expression of the same genes in control mice (represented by the blue bar). Included are BTK and p53, as well as SASP markers (IL‐1B and IL‐6) and markers previously described by us (PLD3, VPS26A and ARMCX3). All values were previously normalized to GAPDH expression. *n* = 4–7. All experiments were done in triplicates. Bars represent mean values, and error bars represent standard deviation. ****p < .0001; ***p = .0002; ns: not significant (unpaired *t* tests). (c) Representative SA‐β‐gal staining of fragments of the brains of two control and two 10 mg/kg ibrutinib‐treated *Zmpste24^−/−^* mice 70 days after the beginning of treatment. (d) Mean number of telomere‐associated foci in CA3 neurons of the hippocampus of control and 10 mg/kg ibrutinib‐treated *Zmpste24^−/−^* mice 121 days after the beginning of treatment. Bars represent average values, and error bars represent standard deviation. *n* = 5. (e) Mean number of γH2A.X foci in the CA3 neurons of the same mice

To test whether BTK inhibition could prevent the age‐dependent buildup of senescent cells in the brain, we measured the expression of different markers of senescence in brains of control and ibrutinib‐treated *Zmpste24^−/−^* mice. Immunostaining confirmed that BTK expression was reduced in treated mice, consistent with previous observations (Althubiti et al., 2016; Rada et al., 2017, 2018a). This was concomitant with a decrease in p53 and p16 levels, as expected (Figure 3a). In line with this, mRNA levels of BTK (which is a transcriptional target of p53 (Althubiti et al., 2016)) and other markers of senescence were also decreased in the brain samples (Figure 3b). p53 mRNA levels did not change significantly, which is compatible with the post‐translational effects of BTK on p53 levels (Althubiti et al., 2016). The reduction in senescent cell accumulation in brains of treated mice was confirmed by a whole organ SA‐β‐gal staining (Figure 3c). Interestingly, there was no change in the percentage of neurons with telomere‐associated DNA damage response foci (TAF; Figure 3d) or histone γH2A.X foci (Figure 3e), both of which are markers of DNA damage that accumulate with age (Jurk et al., 2014). This suggests that BTK inhibitors may prevent the onset of senescence despite the presence of the persistent high levels of DNA damage normally observed in aging and, particularly, in the *Zmpste24^−/−^* mouse model (Varela et al., 2005). These data together show that BTK suppression correlates with a decrease in senescent cell accumulation in the brain of progeroid mice and suggests that the chemical inhibitors could have a potential effect on brain functions.

### 2.4 Effect of BTK inhibitors on anxiety‐like behaviour

**Figure 4:**
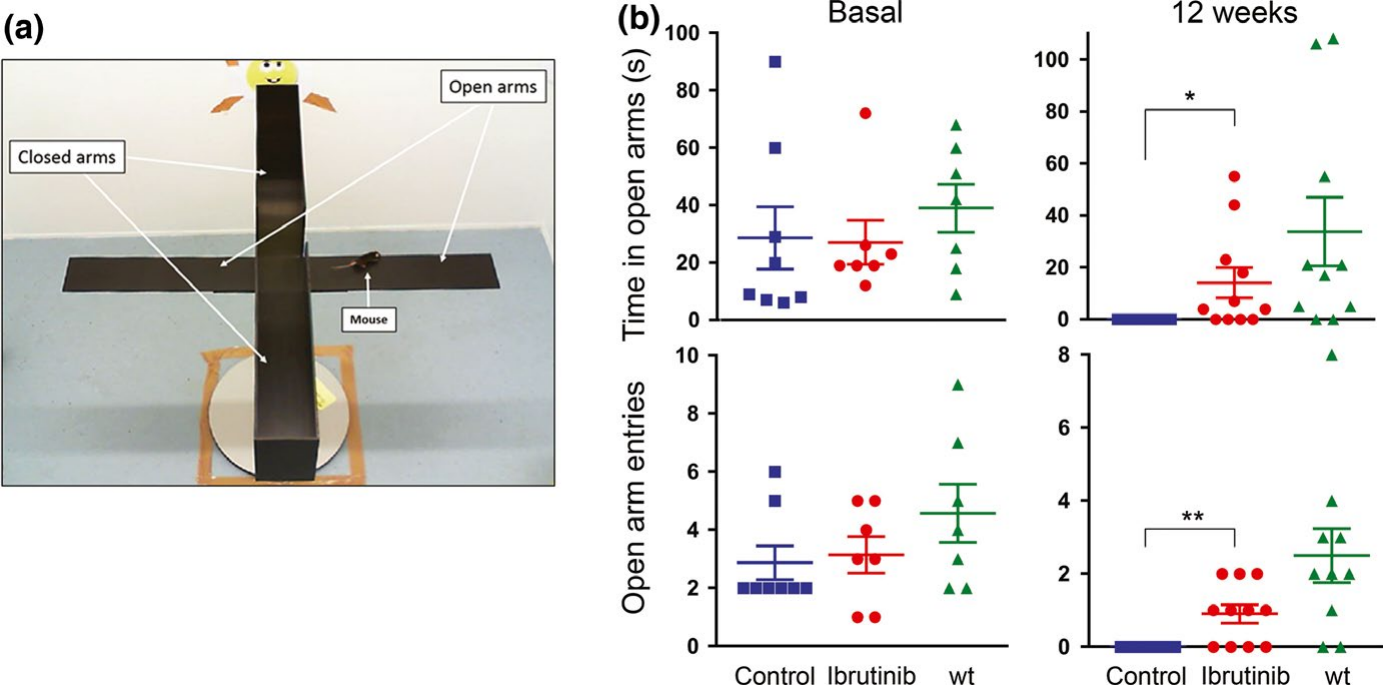
BTK inhibitors reduce anxiety‐like behaviour in *Zmpste24^−/−^* mice. (a) The Elevated plus maze used to assess anxiety‐like behaviour. (b) Average entries and time spent by wild‐type, control and 10 mg/kg ibrutinib‐treated *Zmpste24^−/−^* mice in the open arms of an Elevated plus maze, 12 weeks after the beginning of treatment. Error bars represent standard deviation. *n* = 10 for each group. No significant differences were observed in the basal measurements; *p* = .6 (time) and 0.2 (entries), as measured by one‐way ANOVA. *p = .03; **p = .003 (unpaired *t* tests)

To determine the impact that BTK inhibition may have on brain functions during aging, we performed a series of tests on the ibrutinib‐treated *Zmpste24^−/−^* mice. We first used the elevated plus maze (EPM) to measure anxiety‐like behaviour in mice (Figure 4a), since anxiety has been shown to be strongly associated with aging (Perna, Iannone, Alciati, & Caldirola, 2016) and the presence of senescent cells (Ogrodnik et al., 2019). In the EPM test, we measured the willingness of mice to explore the open arm of the maze, which is inversely proportional to the anxiety experienced (Loxton & Canales, 2017; Walf & Frye, 2007), by counting the number of times a mouse entered into, and the time spent in, the open arms. As shown in Figure 4b, the entries and time spent in open arms were similar in all *Zmpste24^−/−^* mice before starting the ibrutinib treatment (basal). However, 12 weeks into the treatment (by then mice were 6 month old, close to the end of their lifespan), control mice did not venture into the open arms, while the mice receiving ibrutinib still explored them. Of note, treated mice explored less than what wild‐type mice of similar age would do, which confirms the accelerated aging of the *Zmpste24^−/−^* mice. These results suggest that BTK inhibition was able to reduce anxiety‐like behaviour in prematurely aged mice, consistent with an amelioration of age‐related symptoms in a tissue with cells that would normally express high levels of BTK.

### 2.5 BTK inhibition prevents age‐dependent long‐term spatial memory loss

**Figure 5:**
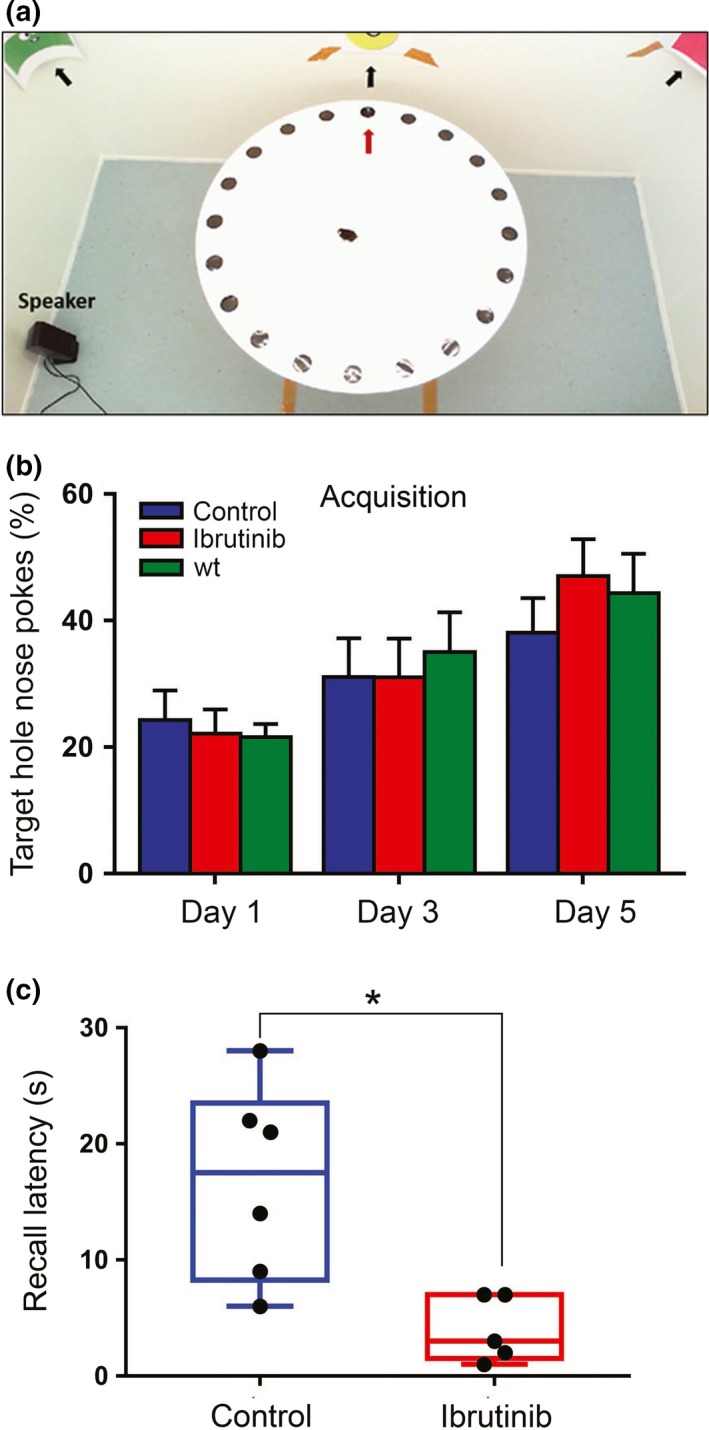
BTK inhibition ameliorates the age‐dependent decline of spatial memory. (a) The Barnes maze set‐up used for cognitive function tests. The target hole is indicated by the red arrow in the figure and fixed visual cues on the wall are indicated by black arrows. A camera positioned on the ceiling recorded the experiments. (b) Percentage of nose pokes that were made on the target hole by mice during the acquisition period of the Barnes maze test, which reflects the ability to learn the task. Control and 10 mg/kg ibrutinib‐treated *Zmpste24^−/−^* mice were compared to wild‐type mice. There were no statistical differences between the three groups in any of the 3 days recorded. *n* = 13 (control) and 15 (ibrutinib). (c) The box and whiskers plot show the recall latency for control and 20 mg/kg ibrutinib‐treated *Zmpste24^−/−^* mice in Barnes maze trials performed 14 days after acquisition, 14 weeks after the beginning of treatment (24 weeks of age). *n* = 6 (control) and 5 (ibrutinib). Statistical significance was determined using the unpaired *t* test. *p = .01

To further explore the impact of BTK inhibition in cognitive functions of old mice, we next used the Barnes maze (Capilla‐Gonzalez et al., 2012) to assess long‐term spatial memory, since memory loss is also associated with aging (Esiri, 2007; Figure 5a). This experiment was chosen because it relies on extra‐maze visual cues and does not use strong aversive stimuli, thereby inducing less stress in the animals than other mazes used for cognitive assessment, such as the radial arm maze and the water mazes (Loxton & Canales, 2017). During the acquisition phase, mice were trained for a week to find a hole that lead to an escape box in a circular surface, which had 19 other holes with no boxes. Control and treated premature aging mice showed similar acquisition curves, also comparable to those of wild‐type mice, which indicates that the *Zmpste24^−/−^* mice had no learning impairment (Figure 5b and S2). However, when the experiment was repeated 2 weeks later, mice treated with 20 mg/kg ibrutinib were significantly faster at recalling the location of the escape box (Figure 5c and Videos S1 and S2). These results together indicate that ibrutinib can prevent the loss of different brain functions and link for the first time BTK expression with age‐related organ decay.

## 3 DISCUSSION

Understanding the biological mechanisms implicated in organismal aging has been a long‐standing priority, given the huge social and economic impact that any strategy to ameliorate it could potentially have. After decades of intensive research, the main processes that determine human aging have begun to be outlined (Lopez‐Otin et al., 2013). This provides a blueprint to devise strategies to slow or delay the age‐dependent functional decay of tissues and organs. Preventing the accumulation of senescent cells, one of the hallmarks of aging that is more amenable to manipulation, can be achieved mainly through two different routes: increasing the clearing of these cells or blocking their emergence. While we previously reported tools that could be used for the former (Althubiti et al., 2014; Althubiti & Macip, 2017; Ekpenyong‐Akiba et al., 2019), in this study we propose a way to influence the latter.

The pathways that lead to cellular senescence have been well studied, and they have been found to mostly converge on the p53‐p21 and the p16‐Rb axes. Dampening the activity of either of them could thus result in a decreased induction of senescence and an eventual reduction in the number of senescent cells present in old tissues. Here, we show a pharmacological intervention, based on our previously published in vitro observations of its effect on the p53 pathway (Althubiti et al., 2016; Rada et al., 2017), that has a significant impact on lifespan and healthspan of mammals.

The obvious downside of this and similar approaches would be that the inhibition of essential tumour suppressor pathways, to which p53 contributes importantly, could increase tumourigenesis. Slowing down the age‐related decline at the cost of increasing the probability of cancer development would be a moot point. However, the possibility that the anti‐tumour and pro‐aging functions of p53‐induced senescence could be uncoupled has been intensively debated. Several mouse models were developed to address this question, with initial reports showing that excessive p53 activity would protect against cancer but lead to premature aging (Maier et al., 2004; Tyner et al., 2002). However, it was later demonstrated that an increased p53 load could actually prevent cancer without increasing aging if it were under the proper control mechanisms (Garcia‐Cao et al., 2002). The converse experiment, eliminating p53 to test whether this increases lifespan, has not been possible due to the fact that cancers inevitably arise early in knockout mice (Harvey et al., 1993). Here, we present the first demonstration that a reduction in p53 activity in mice ameliorates the aging‐related impact on physiological cognitive/affective functions.

To establish the clinical relevance of BTK inhibition in senescence, we chose the *Zmpste24^−/−^* progeroid mouse model for the convenience of a short lifespan, which allows faster and more accessible experiments. Although average lifespan was not increased in this model, we observed differences in parameters linked to healthspan, showing that BTK inhibition indeed had an impact on aging. Importantly, there was no increase in tumourigenesis in our experiments, which suggests that other tumour suppression mechanisms were still capable of protecting these animals despite the impairment in p53‐induced senescence.

The fact that the effects on lifespan were moderate, despite a significant reduction in frailty, suggested that BTK inhibition had a heterogeneous impact in preserving organ function, probably linked to tissue‐specific levels of BTK expression and the role of the p53 pathway on the aging processes of each organ. We are currently performing a detailed exploration of how BTK inhibitors may protect different tissues from aging, which will form the basis for future reports. Here, we focused on the brain, which showed a high increase in BTK expression during aging.

Our results indicate that brain functions that are affected during the aging process may be preserved when BTK activity is blocked, which is consistent with the fact that ibrutinib has been shown to cross the blood–brain barrier (Bernard et al., 2015). This also shows an important role of p53 in the brain aging in this model. The mechanism involved could be a reduction in the presence of senescent cells in the old brain, as suggested by our biochemical analyses, which would be consistent with a recently published report showing that clearance of senescent glial cells prevents cognitive decline in mice (Bussian et al., 2018). However, other senescence‐independent functions of BTK could also be playing a role. Thus, further studies will be needed to fully elucidate the mechanisms involved in the effects of BTK inhibitors on brain aging.

Of note, blocking p53 activation through BTK inhibition does not prevent the accumulation of damage in the neurons normally observed in aging. This shows that although these triggers for senescence are still present, lack of BTK prevents the cell for mounting a full senescent response. It also reinforces the hypothesis that it is not the cellular damage itself but the excessive presence of senescent cells what disrupts tissue homeostasis and activity in aging.

Our study suggests that interfering with the p53 pathway by removing BTK’s regulation could reduce certain features of the aging phenotype without increasing the risk of neoplasia. The fact that inhibition of p53 caused by blocking BTK is not complete (Althubiti et al., 2016) could explain why ibrutinib does not fully recapitulate the phenotype of a p53 null mouse. Other aspects of the impact of BTK inhibition on lifespan and healthspan should also be carefully considered. First, it will be important to reproduce these results in wild‐type mice to assess the role of BTK in normal organismal aging. A careful monitoring of the cancer load in these animals will be crucial to confirm that BTK inhibitors do not interfere with critical tumour suppressor functions. Of note, these drugs are currently being used in the clinic to treat leukaemia and, so far, long‐term follow‐up studies have not reported a significant increase in secondary tumours (Walter et al., 2017). It is possible that blocking p53‐induced senescence through BTK inhibition is not sufficient to disrupt the complex antineoplastic pathways of the cell. For instance, it has been recently proposed that there is an extensive functional overlap of different p53 functions in the context of tumour suppression, thus providing fail‐safe mechanisms should any of them be deregulated individually (Janic et al., 2018). Also, the aging phenotype in normal mice may not be as dependent on the p53 pathway as in *Zmpste24^−/−^* mice, and thus, the effects of BTK inhibitors could be less intense.

It is important to consider that the absence of other regulatory kinases upstream of the p53 pathway has been shown to be embryonically lethal, such as is the case of ATM (Daniel et al., 2012) and ATR (Brown & Baltimore, 2000). However, mice lacking BTK develop normally, although they are severely immunocompromised due to the role of BTK in early B‐cell development (Kerner et al., 1995; Khan et al., 1995). These more limited side effects make BTK a unique target to study the role of the p53 pathway in aging. A conditional knockout model that could switch off BTK at later stages in life could be useful to quantify its impact on aging, since the strong effect that the absence of BTK has on the immune system during development could mask any potential increase in healthspan or lifespan.

It would also be necessary to establish a range of doses of inhibitors that have an effect on different aspects of aging, beyond the ones that we used, which were based on information available for the antileukemic effects of ibrutinib (25 mg/kg is a concentration often used in vivo [Reiff et al., 2018]). Like most targeted inhibitors, ibrutinib is not totally specific and also blocks other kinases. Thus, it cannot be ruled out that some of these off‐target effects contribute to the outcomes we observed. Other BTK inhibitors with higher specificity should be tested in mice to determine whether the effect is maintained. Finally, it would be interesting to determine whether interrupting senescence with BTK inhibition has any effect on lifespan and healthspan if done at later stages of life. In our experiments, these drugs were given to mice continuously from around the beginning of the second third of their lives, what could be the equivalent of the fourth decade in humans. For them to be used to ameliorate aging in humans, it would be convenient if they could be administered following intermittent patterns and/or the treatment could get started at later stages, closer to the last third of life, where the symptoms of aging and loss of brain functions start becoming more evident.

In summary, we have presented evidence that BTK inhibition can have a positive impact on mammalian healthspan in the absence of increased carcinogenesis and that the chemical inhibition of an upstream kinase of the p53 pathway using already clinically approved and well‐tolerated drugs results in an amelioration of the age‐related functional decline in fast‐aging mice. This should accelerate any potential translational applications. An immediate one could be the repurposing of ibrutinib (and other BTK inhibitors currently in clinical trials) to treat progeroid syndromes. Eventually, they may be shown to be useful to prevent senescent cell accumulation in certain situations, such as Alzheimer's disease, as well as reducing the frailty associated with normal aging. However, further experiments will be needed to fully understand the role of BTK in aging and its therapeutic relevance in senescence‐related diseases.

## 4 EXPERIMENTAL PROCEDURES

### 4.1 Senescence‐associated‐β‐galactosidase (SA‐β‐Gal) staining

Freshly extracted brains were stored in −80°C until the time of the experiment. After defrosting, tissues were washed two times with 1× PBS and fixed with 10% neutral‐buffered formalin at room temperature for 15 min. After fixation, the washes in 1× PBS were repeated two times and the 2–3 ml of staining solution (1 mg/ml X‐gal in dimethylformamide [DMF], 40 mM citric acid/Na phosphate buffer pH = 6.0, 5 mM potassium ferrocyanide, 5 mM potassium ferricyanide, 150 mM sodium chloride, 2 mM magnesium chloride in distilled water) was added. Samples were incubated at 37°C in a non‐CO_2_ incubator, and the blue‐coloured β‐galactosidase staining was observed after 24 hr of incubation.

### 4.2 Immunoblot analysis

Brain from humanely culled study animals was cut into small pieces and mashed through a 40‐µm cell strainer (Corning) using the piston of a syringe plunger (Terumo). The tissue homogenate was rinsed out using FBS with 10% DMSO and stored at −80°C for future use. To extract protein from frozen tissue homogenates, 300 µl was taken from each cryovial and washed twice with PBS. Pellets were re‐suspended in RIPA buffer. Samples were incubated on ice for 30 min, after which they were thoroughly lysed by passing through a syringe and 0.8 × 40 mm needle at least 20 times. 1 μg/ml protease inhibitor cocktail set III (Calbiochem) was added to all cell lysates. Protein concentrations were determined using Bradford protein assay (Fermentas). 10–20 µg of total protein per sample was subjected to 10% or 6% SDS PAGE and transferred to immobilon‐P membranes (Millipore) for Western blotting as previously described (Carrera et al., 2014). For antibodies used in Western blots, see Table S3. An ECL detection system (Thermo Scientific) was used to visualize the results. For Ponceau staining, 1× Ponceau staining (Sigma, P 3504) was added to the membrane for 5 min with shaking.

### 4.3 Mouse model of premature aging

In vivo studies were carried out using the zinc metalloproteinase STE24 (ZMPSTE24) deficient mouse model of progeria, which has an average lifespan of 6–8 months (Pendas et al., 2002; Varela et al., 2005). Cryopreserved spermatozoa were purchased from the Mutant Mouse Resource & Research Centre at University of California, Davis, which is supported by the National Institutes of Health (NIH). The *Zmpste24^−/−^* sperm was implanted into recipient female wild‐type C57BL/6J mice. Heterozygous offspring of the founder lines were further bred to create genetically altered strains deficient in the *Zmpste24* gene. All research done using animals were conducted in adherence to the UK Home Office Animals (Scientific Procedures) Act 1986. For humane reasons, animals were allowed to be kept for a maximum of 8 months of age or when a humane end point was reached (see Table S1). All experiments were started when animals reached 2 months of age. *Zmpste24^−/−^* mice received either ibrutinib (dissolved in DMSO) or water with DMSO (controls). Animals from the same group were paired in cages, matched by sex and age. No two animals from the same experimental group were placed into the same cage. Animals were administered either 10 mg or 20 mg of Ibrutinib (PCI‐32765, Selleck) per kilogram body weight, twice weekly by oral gavage, using a 1 ml syringe (Terumo) and a 20 ga × 38 mm plastic feeding tube (Instech Laboratories).

### 4.4 Mouse frailty scoring

A frailty assessment form based on several relevant clinical parameters (Whitehead et al., 2014) was used to score the frailty of the mice after physical examination (performed at least twice weekly). Hearing loss was assessed using a clicker pen; forelimb grip strength was assessed by placing the mouse on its cage grid and gently tugging at its tail; vision was assessed by lifting the mouse and placing them down on the palm of the hand, fore limbs first, to see whether they could put down their paws to support themselves. Parameters were assessed and rated based on the severity as absent, mild or severe. The results were analysed on Microsoft Excel and GraphPad Prism 7.0.

### 4.5 Measurement of anxiety‐like behaviours

A 4‐armed EPM was used to measure anxiety‐like behaviours in mice, as previously described (Lister, 1987; Loxton & Canales, 2017). Briefly, we constructed a maze of black Perspex, elevated 1 m above the floor, which consisted of two arms that were open and two other arms that were closed with black Perspex walls (Pellow, Chopin, File, & Briley, 1985). Mice were placed individually in the centre of the EPM and allowed 5 min to explore it. The mice's entries into open and closed arms and the time spent in each set of arms were recorded. The results were manually analysed by a blinded observer.

### 4.6 Long‐term spatial memory assessment

A Barnes maze was used to determine spatial memory in mice, as described (Barnes, 1979). Briefly, a white circular platform raised 1 m above the ground had 20 equidistant holes around its edges, with only one of them with a black escape box fitted underneath (target hole). White noise was played on speakers, while a mouse was placed in the centre of the platform under bright light. Visual cues and the position of the target hole were kept constant throughout the study. Acquisition (training) was carried for 5 days after an initial habituation session. Fourteen days after acquisition, the experiment was repeated with the escape box removed the hole sealed like all the others. The latency (time to locate the target hole) and number of nose pokes into the target hole (compared to those into the wrong holes) were calculated. Two blinded observers manually analysed the data obtained.

### 4.7 Immunostaining

Brains were isolated from mice that were culled humanely and immediately placed into specimen collection tubes (Sterlin, No. 25052B) containing 10% neutral‐buffered formalin (Sigma‐Aldrich) and fixed for 24–48 hr. Tissues were removed from formalin and placed in 70% ethanol before being processed into paraffin‐embedded tissue blocks. Formalin‐fixed paraffin‐embedded (FFPE) tissues were cut into 5‐µm sections using a microtome (LEICA RM2235). Sections were then placed on a 34°C tissue floatation bath (RA Lamb) to straighten out before being mounted on slides. Slides were previously coated with a solution of 3‐aminopropyltriethoxysilane, as described (Maddox & Jenkins, 1987), to enhance tissue adhesion. Slides were either air‐dried overnight or dried in the oven at 37°C for 1 hr and then dewaxed in two changes in xylene for 10 min each. They were then dehydrated in two changes of 100% ethanol for 10 min each, after which the slides were soaked in 400 ml of methanol containing 2.4 ml of 30% hydrogen peroxide for 10 min, to block endogenous peroxidase. Slides were thereafter microwaved for 15 min in citrate buffer in a pressure cooker for heat‐mediated antigen retrieval and then washed with 1× PBS. The edges of the slides were dried, and a pap pen was used to mark around the tissue. Slides were incubated with 100 µl of 5% serum for 1 hr, after which the serum was drained, and slides were then incubated with primary antibody overnight at 4°C. The slides were washed in PBS, and the edges dried before incubating for 30 min with streptavidin‐peroxidase. They were again washed and incubated with DAB (3,3′‐diaminobenzidine) substrate and peroxidase for 5 min maximum. Slides were washed then dehydrated in 70%, 90% and 100% ethanol for 10 min each. For antibodies used, see Table S3. Quantification (intensity of staining expressed as the percentage of DAB signal in a field) was done using immunoratio (http://153.1.200.58:8080/immunoratio/).

### 4.8 Immunostaining and telomere‐associated foci (TAF) and quantification

Paraffin sections were deparaffinized with histoclear and hydrated in an ethanol gradient followed by water and PBS. Antigen was retrieved by incubation in 0.01 M citrate buffer (pH 6.0) at 100°C for 10 min. Slides were placed in blocking buffer (1:60 normal goat serum [S‐1000, Vector Laboratories] in 0.1% BSA/PBS) for 60 min at room temperature. For TAF staining, slides were additionally blocked with avidin/biotin (Vector Lab, #SP‐2001) for 15 min each. Primary antibodies used (γH2A.X, table below) were diluted 1:250 in blocking buffer and applied overnight at 4°C. The next day, slides were washed three times with PBS and incubated for 30 min with secondary goat, anti‐rabbit antibody (1:200; Vector Laboratories #BA‐1000). Fluorescein–avidin in PBS (1:500; #A‐2011, Vector Lab) was applied to each sample for 20 min. Slides were washed three times in PBS, which was followed by FISH for TAF detection. Briefly, tissues were crosslinked with 4% paraformaldehyde for 20 min and dehydrated in graded ethanol. Sections were denatured for 10 min at 80°C in hybridization buffer (70% formamide (Sigma), 25 mM MgCl_2_, 0.1 M Tris (pH 7.2) and 5% blocking reagent [Roche]) containing 2.5 μg/ml Cy‐3‐labelled telomere‐specific (CCCTAA) peptide nucleic acid probe (Panagene), followed by hybridization for 2 hr at room temperature in the dark. Slides were washed twice with 70% formamide in 2× SSC for 15 min, followed by washes in 2× SSC and PBS for 10 min. Sections were mounted in Vectashield DAPI‐containing mounting media and imaged. Anti‐γ‐H2A.X rabbit monoclonal antibody: Cell Signaling Technology # 9718; RRID: AB_2118009.

### 4.9 Quantitative real‐time PCR

Total RNA was extracted from FFPE mouse brain tissues using the ReliaPrep™ FFPE Total RNA Miniprep System Kit (Promega) and following manufacturers’ instructions. The concentration of RNA was measured using a NanoPhotometer P300 (Implen). Samples were used for cDNA synthesis using the Superscript™ III First‐Strand Synthesis System for RT‐PCR (Invitrogen), following manufacturer's instructions. Quantitative real‐time PCR was performed in a LightCycler^®^ 480 Multiwell Plate 96 (Roche) using SensiMix SYBR No‐Rox (Bioline), in triplicates, as previously described (Althubiti et al., 2016). For the primers used, see Table S4. All primers were supplied by Eurofins Scientific. A melting curve was used to prove the specificity of the primers at the end of the PCR run. Quantification was done with the ∆∆C_t_ method. Results were analysed on Microsoft Excel, and graphs plotted using GraphPad Prism 7.0 Software.

## CONFLICT OF INTEREST

The authors declare no conflicts of interest.

## AUTHORS’ CONTRIBUTIONS

Experiments were designed by SM and AEEA and performed by AEAA with MP, MA and MR. SM, AEEA and MP analysed the data and prepared the figures. SG and GKF helped with mouse experiments. JJC contributed to the design and interpretation of cognitive tests. YS contributed to analyse the results. TAF and γH2A.X foci measurements were designed and performed by DJ. SM and AEEA wrote the manuscript and all authors read it.

## Data Availability

Data will be made available through the university's public databases. The University of Leicester launched its Figshare for data institutional platform in Summer 2018 (https://leicester.figshare.com/). Arkivum was procured and installed as a safe, secure, compliant and accessible digital archiving solution for research data outputs underlying the institutional repository. Arkivum is fully certified and audited to the ISO 27001:2013 Standard in Information Security Management. The University of Leicester research data management policy will store and preserve research data outputs for a minimum of 10 years.
