# Supplementary material for: Amelioration of age‐related brain function decline by Bruton's tyrosine kinase inhibition
Source: Aging Cell. 2019 Nov 17;19(1):e13079. doi: 10.1111/acel.13079 (PMC6974713; doi:10.1111/acel.13079)
Supplement: Supplementary file 1 [file ACEL-19-e13079-s001.docx]

**SUPPLEMENTARY FIGURES**


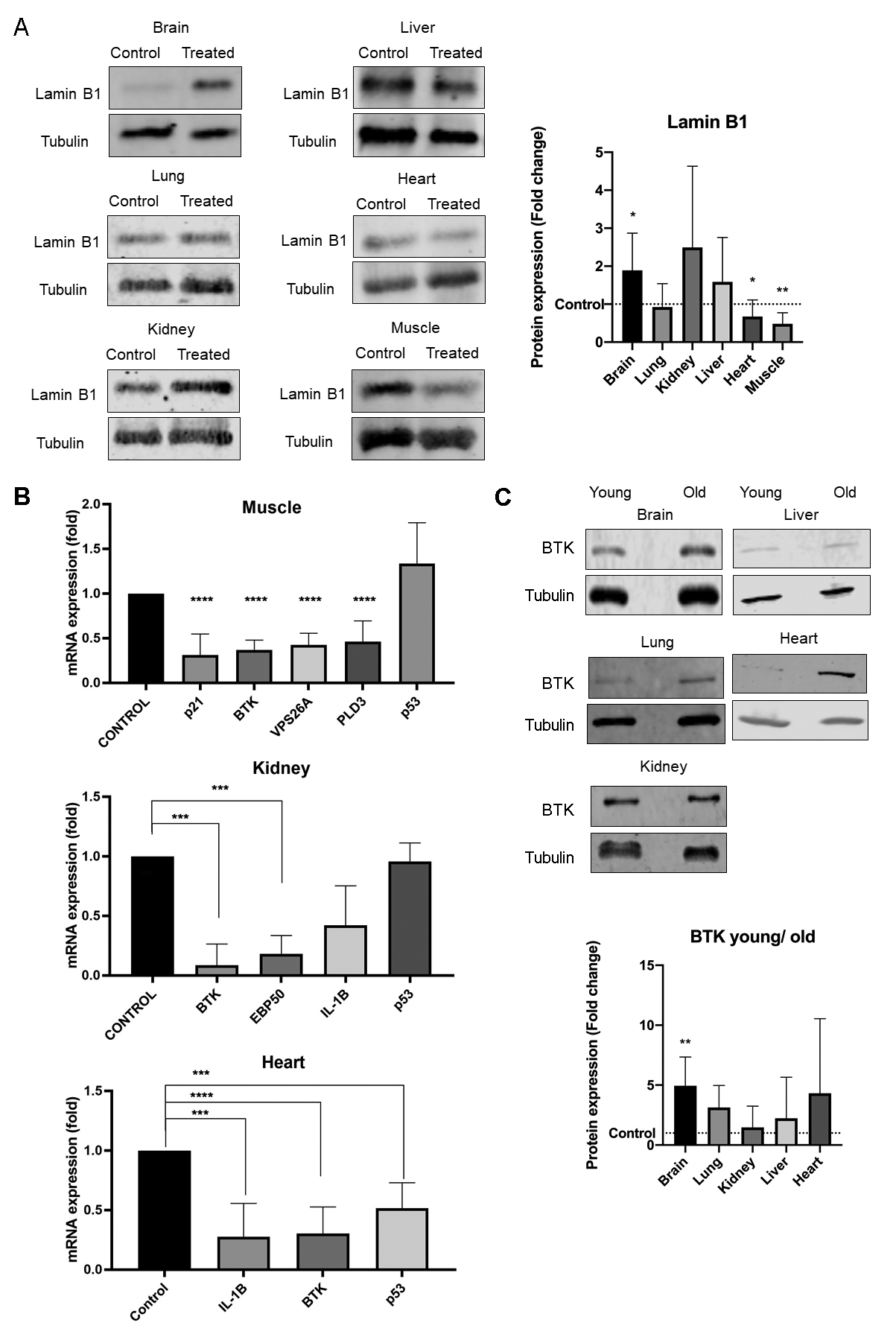


**Supplementary Figure 1. Ibrutinib reduces the presence of senescent cells in tissues of *Zmpste24^-/-^* mice. (A)** Representative Western blots showing the expression of Lamin B1 in brain, liver, lung, heart kidney and muscle, as compared to a loading control (tubulin). The average of three independent experiments is plotted in the graphic, normalizing the expression of Lamin B1 to that of tubulin and the controls. **(B)** Quantitative real time PCR of the same samples, measuring the expression of different markers of senescence in ibrutinib-treated mice, and normalizing to the expression in control mice. **(C)** Representative Western blots showing expression of BTK (top) and tubulin (bottom) in brain, liver, lung, heart and kidney. The average of three independent experiments is plotted in the graphic, normalizing the expression to tubulin and the controls. All error bars represent standard deviation. * p<0.03; ** p<0.003; *** p<0.0003; **** p<0.0003 (unpaired t tests).


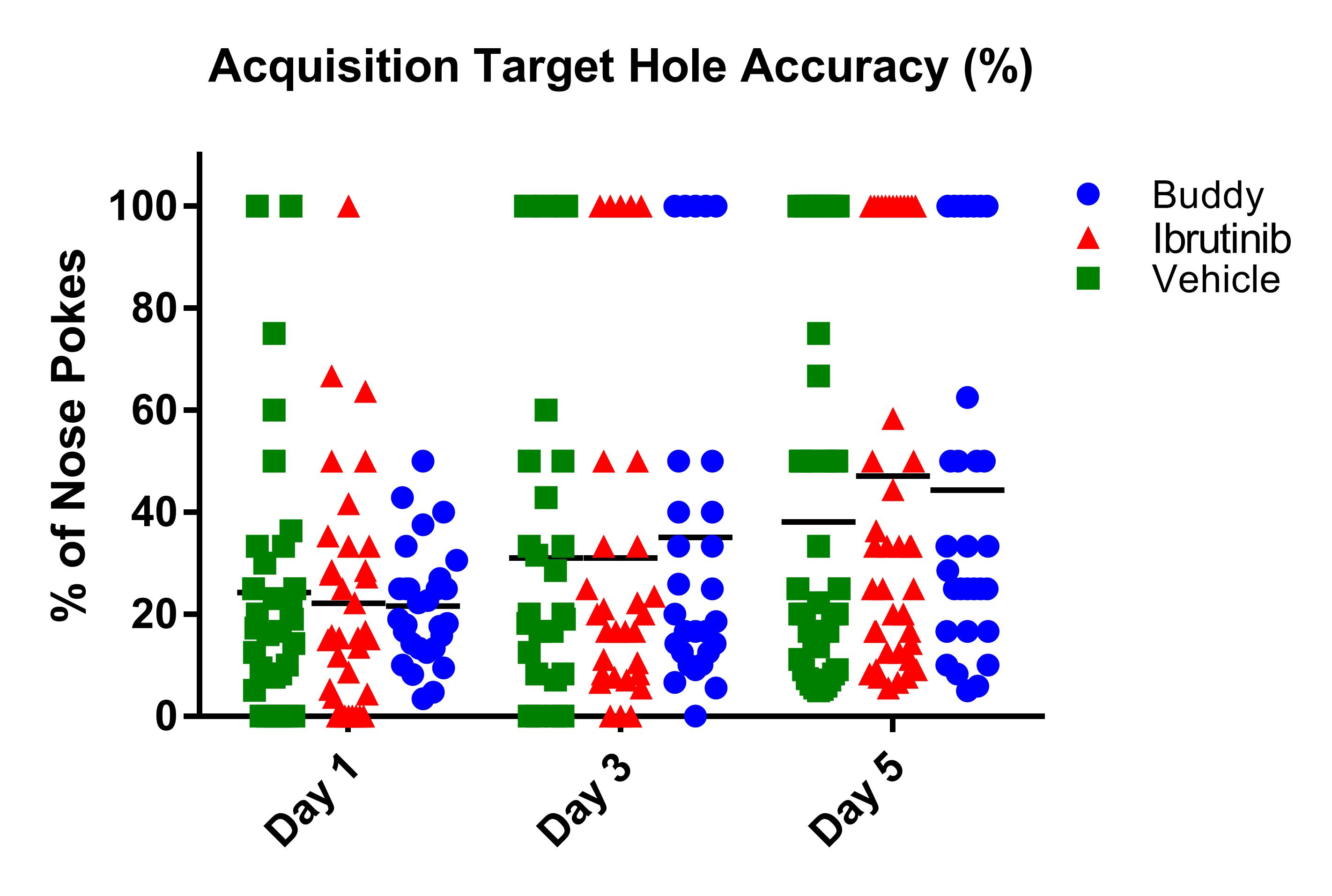


**Supplementary Figure 2. Barnes Maze on target nose poke percentages.** Same data as in Figure 5B, presented here in dot plots to better assess individual variability. Each dot represents a measurement from one of the mice in the cohort and the horizontal bars represent the averages.

**Supplementary Videos.** Representative recordings of control (#1) and 20 mg/kg ibrutinib-treated (#2) *Zmpste24^-/-^* mice undergoing a recall test in the Barnes Maze.

**SUPPLEMENTARY TABLES**

| **Parameters** | | **Score** |
| --- | --- | --- |
| Appearance | - Normal  - Slight piloerection  - Marked piloerection  - Changes from normal to signs of deterioration of coat and skin | 0  1.5  3  4-6 |
| Natural behaviour | - Normal  - Minor changes  - Less mobile and isolated  - Restless or very still | 0  1  2  3-6 |
| Food and water intake | - Normal  - Body weight loss <5%  - Body weight loss <10% - < 20% | 0  3  4-6 |
| Hydration status | - Normal  - Abnormal skin pinch test  - Very Abnormal skin pinch test | 0  2.5  2.5-6 |
| Body changes | - Normal  - Backbones visible  - Backbones very visible  - Tachypnoea (fast breathing)  - Dyspnoea (difficult breathing) | 0  3  6  3  6 |
| Locomotion | - Slightly abnormal gait/posture  - Markedly abnormal gait/posture | 0-1.5  3-6 |
| Muscle tone | *-* Muscle groups have normal tone or mass  - Muscle mass slightly soft  -Muscle mass less firm, abdomen slightly soft  - Muscle mass very thin, soft, undefined  - Muscle mass has no tone or definition | 0  2  3  4  5-6 |
| Specific Indicators | - Tumour size >1.0cm^3^  - Tumour impeding movement | 6  6 |

**Table 1.** Distress Scoring Sheet used for monitoring progression of clinical signs in mice and defining the experimental end point. Scores for all parameters assessed were added every time the mice were checked and the following actions taken depending on the total value:

0-3: no action.

3-6: monitor carefully (including regular health checks), inform PIL and consider intervention, if necessary consult NACWO and/or NVS, provide intervention pain relief/fluids etc. as appropriate/advised.

≥6: terminate using schedule 1 method (humane end point).


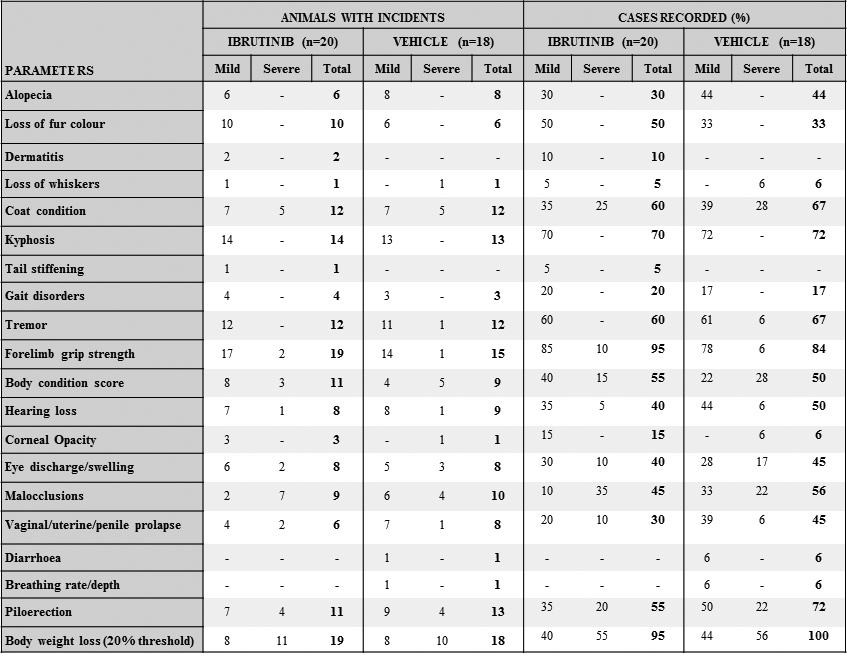


**Table 2.** Clinical frailty score list used for the Zmpste24^-/-^ mice experiments.

| Name | Source | Clonality | Application | Dilution | Supplier / Product No. |
| --- | --- | --- | --- | --- | --- |
| β-actin | Rabbit | Polyclonal | WB | 1:5,000 | Abcam, Ab8227 |
| BTK | Rabbit | Monoclonal | WB, IHC | 1:500, 1:100 | Cell Signaling, (D3H5) #8547S |
| p16 | Mouse | Monoclonal | IHC | 1:500 | Abcam, Ab54210 |
| p53 | Rabbit | Polyclonal | IHC | 1:100 | Santa Cruz, (FL-393) sc-6243 |
| DEP1 | Rabbit | Polyclonal | IHC | 1:100 | Bioss, bs-2567R |
| IL-6 | Rabbit | Polyclonal | IHC | 1:100 | Boster- PB9034 |
| B2M | Rabbit | Polyclonal | IHC | 1:500, 1:100 | Abcam, Ab87483 |
| Lamin B1 | Rabbit | Polyclonal | WB | 1:500 | Abcam, Ab16048 |

**Table S3.** List of antibodies and dilutions used in Western Blots (WB) and Immunohistochemistry (IHC) from mouse tissues.

| Gene | Primer sequence (5′🡪 3′) |
| --- | --- |
| B2M | Fwd: GCTATCCAGAAAACCCCTCAA |
|  | Rev: CATGTCTCGATCCCAGTAGACGGT |
| ARMCX3 | Fwd: CTGGAGCCTGCTATTGCATTT |
|  | Rev: TCAGACCAGTCATTATACCTGGC |
| p53 | Fwd: CACAGCGTGGTGGTACCTTA |
|  | Rev: TCTTCTGTACGGCGGTCTCT |
| BTK | Fwd: ACAGATTCCGAGGAGAGGTGAGG  Rev: GGTCCTTCATCATATACAACCTGGAATGG |
| PLD3 | Fwd: CTGAGGAACCGGAAGCTGT  Rev: GGAAAGGGGTGGTCCTGA |
| VPS26A | Fwd: CTGGAAAGAGGCTAGAGCATCA  Rev: AGGCAAGGCTAGTTCCTTCAC |
| STX4 | Fwd: GGTGTCAAGTGTGAGAGAG  Rev: AACCTCATCTTCATCGTCTG |
| IL-1B | Fwd: TCCAGGATGAGGACATGAGCAC  Rev: GAACGTCACACACCAGCAGGTTA |
| IL-6 | Fwd: CCACTTCACAAGTCGGAGGCTTA  Rev:  GCAAGTGCATCATCGTTGTTCATAC |
| GAPDH | Fwd: GTTGTCTCCTGCGACTTCA |
|  | Rev: GGTGGTCCAGGGTTTCTTA |

**Table S4.** List of primers used for quantitative real time PCR.
